# Assessment of Lung Recruitment by Electrical Impedance Tomography and Oxygenation in ARDS Patients

**DOI:** 10.1097/MD.0000000000003820

**Published:** 2016-06-03

**Authors:** Long Yun, Huai-wu He, Knut Möller, Inéz Frerichs, Dawei Liu, Zhanqi Zhao

**Affiliations:** From the Department of Critical Care (YL, H-wH, DL), Chinese Academy of Medical Sciences, Peking Union Medical College Hospital, Beijing, China; Institute of Technical Medicine (KM, ZZ), Furtwangen University, Villingen-Schwenningen; and Department of Anesthesiology and Intensive Care Medicine (IF), University Medical Center of Schleswig-Holstein Campus, Kiel, Germany.

## Abstract

We hypothesized that not all patients with appreciably recruited lung tissue during a recruitment maneuver (RM) show significant improvement of oxygenation. In the present study, we combined electrical impedance tomography (EIT) with oxygenation measurements to examine the discrepancies of lung ventilation and perfusion versus oxygenation after RM.

A 2-minute RM (20 cm H_2_O positive end-expiratory pressure [PEEP] + 20 cm H_2_O pressure control) was prospectively conducted in 20 acute respiratory distress syndrome patients from January 2014 to December 2014. A decremental PEEP trial was performed to select the PEEP level after RM. A positive response to RM was identified as PaO_2_ + PaCO_2_ ≥400 mm Hg. Relative differences in the distribution of ventilation and perfusion in the most dependent region of interest (ROI4) were monitored with EIT and denoted as the ventilation-perfusion index.

Ten patients were found to be responders and 10 patients to be nonresponders. No significant difference in baseline PaO_2_/FiO_2_ was observed between nonresponders and responders. A significantly higher PaO_2_/FiO_2_ ratio during RM and higher PEEP set after PEEP titration were recorded in responders. In both responders and nonresponders, the proportion of ventilation distributed in ROI4 compared with the global value was lower than the cardiac-related activity before RM, but this situation was reversed after RM (*P* < 0.01 in each group). Six out of 10 nonresponders exhibited a remarkable increase in ventilation in ROI4. A significant difference in the relative ventilation-perfusion index was found between the patients with remarkable and insufficient lung tissue reopening in the nonresponder group (*P* < 0.01).

A discrepancy between lung tissue reopening and oxygenation improvement after RM was observed. EIT has the potential to evaluate the efficacy of RM by combining oxygenation measurements.

## INTRODUCTION

Acute respiratory distress syndrome (ARDS) is a life-threatening medical condition with the hallmark of severe hypoxemia.^1^ Patients with ARDS require an intensive care unit (ICU) stay and mechanical ventilation support. The application of a recruitment maneuver (RM) and positive end-expiratory pressure (PEEP) is aimed at opening the collapsed diseased pulmonary areas, subsequently maintaining the atelectatic areas in an open state, and thereby reducing the risk of hypoxemia.^2^ During RM, the peak inspiratory pressure is raised to a high level for a period of time (eg, 40 cm H_2_O or above for 10 breaths or longer) to open the lung.^3^ Various forms of RM have been proposed to optimize this procedure (to recruit more areas and induce less barotrauma).^4–6^ However, the efficacy of RM depends on an individual's lung recruitability.^7,8^ This recruitability, often assessed via computed tomography (CT), increases with ARDS severity and is associated with ICU mortality.^8^ Unfortunately, the application of CT for bedside monitoring is limited due to the risks of radiation exposure in patients. Borges et al^6^ proposed the assessment of RM efficacy by measuring the sum of the partial pressures of oxygen and carbon dioxide in the arterial blood (PaO_2_ + PaCO_2_). Lowhagen et al^9^ suggested combining electrical impedance tomography (EIT) with measurement of the end-expiratory lung volume (EELV) and respiratory system compliance to determine the potentially recruitable lung volume.

Electrical impedance tomography is a noninvasive and radiation-free technique. It uses a set of electrodes that are attached equidistantly around the thorax while small imperceptible alternating currents are injected, and the resultant voltages are measured. Subsequently, relative impedance changes are reconstructed in the measurement plane.^10^ EIT has the potential to monitor regional lung aeration and dynamically visualize the regional distribution of ventilation at bedside.^11,12^ The reliability of EIT has been proven in a number of studies.^13–15^ Several studies have shown that EIT is able to characterize the efficacy of RMs.^16–20^

Previous studies have focused on discussing the recruitability of lung tissues and the pros and cons of RMs.^6,7,21,22^ The aim of RM is to improve oxygenation and minimize ventilator-associated lung injury (VALI). However, there are no studies showing that the amount of recruited lung tissue and the improvement of oxygenation are linearly correlated in individual patients. Patients may benefit from a higher PEEP because their lungs experience mechanical recruitment and become less prone to the development of VALI. However, this effect may not lead to a satisfactory improvement of oxygenation. We hypothesized that not all patients exhibiting remarkably recruited lung tissue during RM show significant improvement of oxygenation and vice versa. Because EIT can be used to measure the increase in aeration during tidal ventilation, which indicates the amount of recruited lung tissue, we combined EIT with oxygenation measurements to examine the discrepancies of lung ventilation and perfusion versus oxygenation during RM.

## METHODS

### Patients and Protocol

Acute respiratory distress syndrome patients subjected to mechanical ventilation in the Department of Critical Care Medicine in the Peking Union Medical College Hospital were included in this prospective study. They were admitted to the ICU within 72 hours after the onset of ARDS and required mechanical ventilation due to their oxygenation conditions. RMs and PEEP titrations were scheduled as part of the treatments. The exclusion criteria were as follows: age <18 years, patients who were pregnant or within the lactation period, and any contraindication to the use of EIT (eg, a pacemaker, automatic implantable cardioverter defibrillator, or implantable pump). The study was approved by the Ethics Committee of the Peking Union Medical College Hospital. Written informed consent was obtained from all patients or their legal representatives before the study.

Every patient was ventilated in the supine position with an Evita 4 ventilator (Dräger Medical, Lübeck, Germany) using the following initial settings. The tidal volume (*V*_T_) was set to 6 ml/kg ideal body weight. The fraction of inspired oxygen (FiO_2_) and PEEP were adjusted to maintain peripheral capillary oxygen saturation (SpO_2_) over 90% (IntelliVue MP70, PHILIPS, Amsterdam, the Netherlands). If the plateau pressure (*P*_plat_) was >30 cm H_2_O, *V*_T_ was decreased by 1 mL/kg gradually until *P*_plat_ was <30 cm H_2_O or *V*_T_ was <4 mL/kg ideal weight. All of the patients received deep sedation to suppress spontaneous breathing at baseline.

After 10 to 15 minutes of baseline ventilation, PEEP was switched to a zero end-expiratory pressure (ZEEP), and FiO_2_ was increased to 100% for 2 minutes. Subsequently, a 2-minute RM (20 cm H_2_O PEEP + 20 cm H_2_O PC) was performed. Arterial blood gases were examined after RM, and FiO_2_ was then rapidly decreased to maintain SpO_2_ in the range of 93% to 95% over 10 minutes. Subsequently, a decremental PEEP trial was initiated. PEEP was decreased from 20 cm H_2_O in steps of 3 cm H_2_O every 5 minutes. If SpO_2_ fell by more than or equal to 1% to 2% during the decremental PEEP trial, RM was performed again, and PEEP was subsequently set to the level before the drop in SpO_2_.

### Data Collection and Analysis

Blood gas measurements were performed with an ABL90 FLEX analyzer (Radiometer Medical ApS, Br⊘nsh⊘j, Denmark). Dynamic respiratory system compliance (Crs) was measured by the ventilator. An EIT electrode belt with 16 electrodes was placed around the thorax in the fifth intercostal space, and 1 reference electrode was placed at the patients’ abdomen (PulmoVista 500, Dräger Medical, Lübeck, Germany). Electrical alternating currents were applied in a sequential rotating process through adjacent electrode pairs. The resulting differences in surface potential between neighboring electrode pairs were measured. The stimulation frequency and amplitude were adjusted automatically by the EIT device to minimize the influence of background noise. EIT measurements were continuously performed at 20 Hz from baseline through RM to the decremental PEEP trial. Corresponding EIT data were recorded. For EIT data reconstruction, a finite element method-based linearized Newton-Raphson reconstruction algorithm was employed, using the software provided by the EIT manufacturer (Dräger EIT Data Analysis Tool 6.1, Dräger, Germany). The baseline of the images referred to end-expiration at ZEEP.

A tidal image was calculated as the difference between the EIT images at end-inspiration and end-expiration for a tidal breath, which represents the regional distribution of *V*_T_ (the tidal variation of impedance). Five consecutive breaths at the end of each PEEP step were selected, and a mean tidal image was calculated. The highest pixel value in the mean tidal image was identified. Subsequently, pixels with a value greater than 20% of the highest pixel value were defined as ventilated lung regions at the corresponding PEEP. Because ventilated lung regions change during PEEP titration, the estimated lung regions were defined by combining the ventilated lung regions recorded at all PEEP steps (logical OR operation). The tidal images were divided into regions of interest (ROIs) of four anteroposterior segments with equal height.

The global inhomogeneity (GI) index, indicating the degree of homogeneity of the ventilation distribution, was introduced in previous studies.^11,23^ Applying Eq. 1, the GI index was calculated for the five consecutive breaths at the end of each PEEP step.
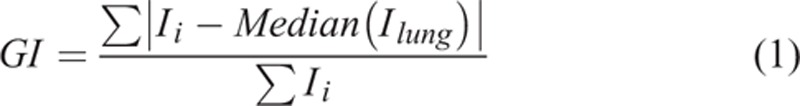


where *I*_*i*_ denotes the impedance value of pixel *i* in the identified lung regions (*i*εlung), and *I*_lung_ represents all pixels in the lung regions under observation.

The center of ventilation (CoV) index indicates the vertical distribution of ventilation.^18,24^ The CoV index was also calculated for the five consecutive breaths at the end of each PEEP step, using Eq. 2.
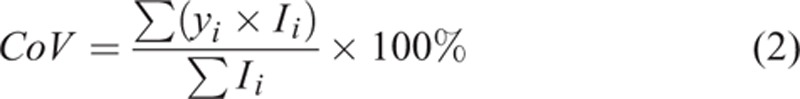


where *y*_*i*_ is the pixel height of pixel *i* scaled so the most ventral row is 0% and the most dorsal row is 100%.

Lung perfusion was estimated by analyzing the cardiac-related EIT signal.^25,26^ The EIT signals were band-pass filtered (Dräger EIT Data Analysis Tool 6.1, Dräger, Germany), with the cut-off frequency varying according to the patients’ heart rates. Due to the phase shifts of cardiac-related signals in different regions (eg, lungs, heart) and the limited signal-to-noise ratio, the standard deviations (SDs) of the cardiac-related signals were calculated pixel-by-pixel to represent the level of cardiac-related impedance changes. The ratio of SD in the most dependent ROI4 to the global value was calculated in each patient. The EIT-based relative ventilation-perfusion index in ROI4 was defined as follows:
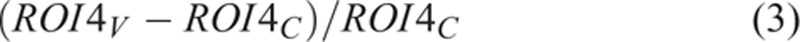


where ROI4_V_ is the proportion of ventilation in ROI4 as a percentage, and ROI4_C_ denotes the proportion of the cardiac-related signal in ROI4 as a percentage. The EIT-based ventilation-perfusion index after RM was also calculated for ROIs 1 to 3 using Eq. 3. The results are reported in percentages.

We defined the following criteria for the study: patients were diagnosed as responders to RM when the value of PaO_2_ + PaCO_2_ was ≥400 mm Hg and as nonresponders otherwise. This criterion identified functional recruitment using oxygenation. The regional tidal variation in ROI4 proportional to the global value as a percentage was calculated after RM (at the highest PEEP) and before RM (at ZEEP). The difference was denoted as ΔROI4. The natural logarithms of ΔROI4 were calculated to magnify the small increase in ventilation in ROI4. We defined remarkable mechanical recruitment as a natural logarithm of ΔROI4 higher than 1 in patients. Otherwise, the outcome of RM was denoted as mechanically insufficient. Thus, this criterion identified mechanical recruitment by assessing lung tissue reopening.

### Statistical Analysis

Data analysis was performed using MATLAB 7.2 (The MathWorks Inc., Natick, MA). Normal distribution of the data was confirmed with the Lilliefors test. Results were expressed as mean values ± SD. A 2-sample *t* test was applied to assess the differences in various parameters measured in responders and nonresponders. A *P* value <0.05 was considered statistically significant.

## RESULTS

A total of 20 consecutive ARDS patients were included. Their demographic information is reported in Table 1. There were 16 (80%) cases of pneumonia, 2 (10%) cases of multiple trauma, and 2 (10%) cases of severe acute necrotic pancreatitis. Patients were classified as showing severe, moderate, or mild ARDS according to the Berlin Definition,^27^ and all patients received a bedside echo-cardiograph to exclude cardiogenic factors. Ten patients were found to be responders and 10 patients to be nonresponders based on oxygenation. Their baseline data are summarized and compared in Table 2. Most of the parameters were comparable between these groups, except that a significantly lower mean arterial pressure was observed in nonresponders. There was no significant variation of the response of blood pressure (defined as a decrease to less than 20% of the baseline value) to RM in all patients.

**TABLE 1:**
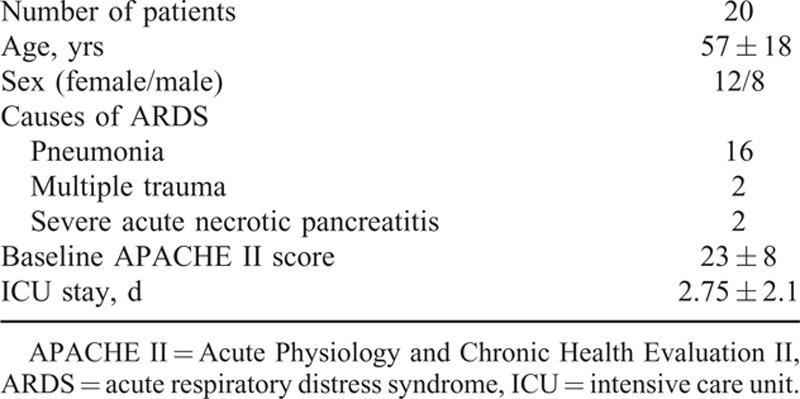
Demographics of the Study Population

**TABLE 2:**
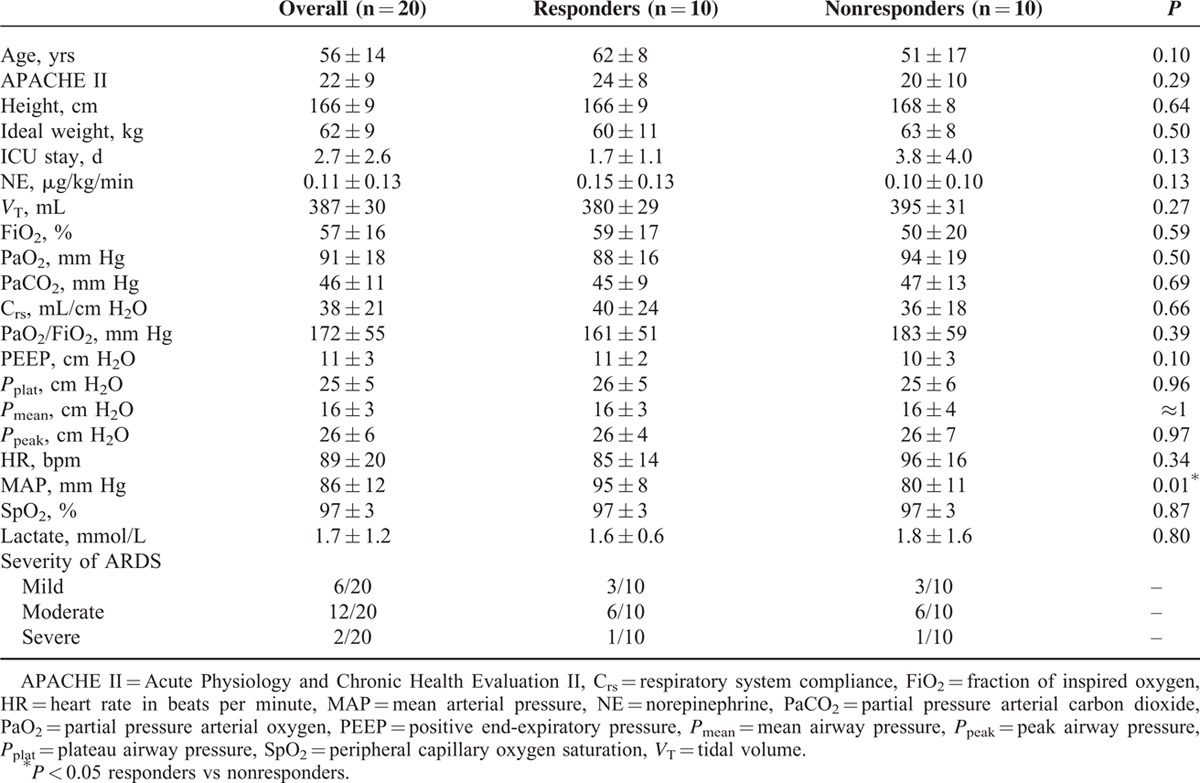
Baseline Data Comparison

The PaO_2_ was higher in the responders than in nonresponders during RM (401 ± 36 vs 300 ± 52 mm Hg; *P* < 0.001; FiO_2_ was set to 100%). No significant differences in Crs, central venous pressure, or various airway pressures were found between the 2 groups. The PEEP set after titration was higher in responders than in nonresponders (12 ± 2  vs 10 ± 2 cm H_2_O; *P* < 0.01).

**FIGURE 1:**
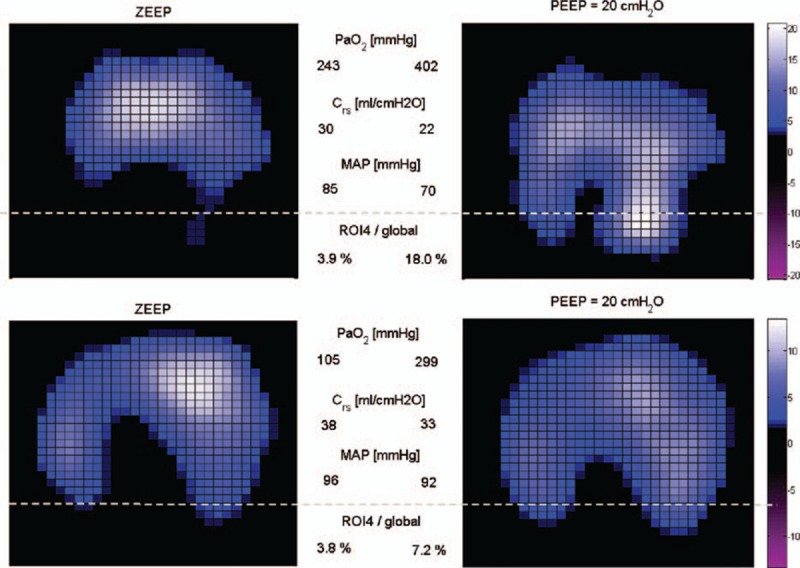
Tidal variation (TV) of 2 patients. One patient was classified as a responder (top), and the other was a nonresponder (bottom) based on oxygenation. Left: patients were ventilated with a zero end-expiratory pressure (ZEEP). Right: patients were ventilated with a positive end-expiratory pressure (PEEP) of 20 cm H_2_O (recruitment maneuver). Highly ventilated regions are coded in light blue. Pixel values are the relative impedance in arbitrary units. The dashed line demarcates the most dependent region (ROI4) in the EIT image. C_rs_ = respiratory system compliance, EIT = electrical impedance tomography, MAP = mean arterial pressure, PaO_2_ = arterial partial pressure of oxygen, ROI4/global = regional TV as a % of global TV occurring in ROI4.

**FIGURE 2:**
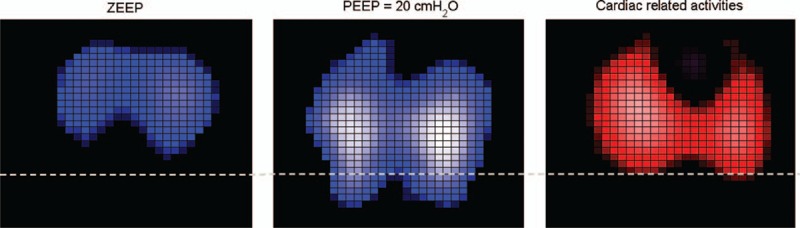
Tidal variation (TV) of a patient classified as a nonresponder (left and middle) and the corresponding functional image indicating cardiac activities (right). Highly ventilated regions are coded in light blue (left and middle sub-figures). Regions with high cardiac activities are coded in light red. The dashed line demarcates the most dependent region (ROI4) in the EIT image. EIT = electrical impedance tomography, ROI = region of interest.

Figure 1 shows tidal images from 1 responder and 1 nonresponder at ZEEP and PEEP of 20 cm H_2_O. Remarkable mechanical recruitment (lung tissue reopening) was observed in ROI4 of the responder (Figure 1, top), whereas moderate lung recruitment was observed in ROI4 of the nonresponder (Figure 1, bottom). Figure 2 shows tidal images from another patient who was classified as a nonresponder (PaO_2_ + PaCO_2_ value of only 297 mm Hg at PEEP of 20 cm H_2_O after RM). In the last patient, lung recruitment was evident (regional TV in ROI4 rose from 3.6% to 11.6%, and the CoV index rose from 40.0% to 53.4%). Because the heart rate of this patient was approximately 110 beats per minute, a band-pass filter with a cut-off frequency of 90 to 150 Hz was used to determine cardiac-related activities. The mean differences in peak-to-peak impedance are presented in Figure 2 (right). Little lung perfusion was evident in ROI4.

**FIGURE 3:**
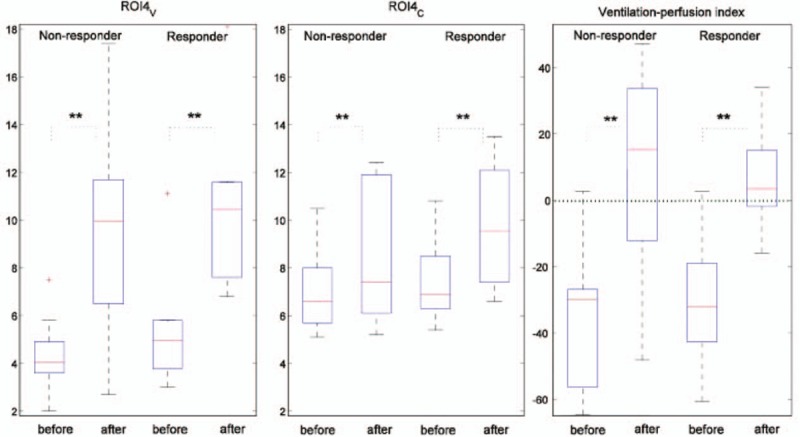
Ventilation distribution (left) and cardiac-related activities (middle) in ROI4 before and after recruitment maneuvers. Nonresponders and responders were divided based on the oxygenation observed after the recruitment maneuver. The relative ventilation-perfusion index (right) was calculated based on Eq. 3. The green dotted line represents the 0 baseline, at which ROI4V and ROI4C matched in proportion to respective global values. All plotted values are percentages (%). ^∗∗^indicates significant differences within each group (*P* < 0.01). The boxes represent the quartiles, whereas the whiskers extend from the box out to the most extreme data value within 1.5× of the interquartile range of the sample. ROI = region of interest.

The ventilation distribution and cardiac-related activities in ROI4 are summarized in Figure 3. In the 10 nonresponders, the ventilation distribution in ROI4 (ROI4_V_) was slightly lower than in the 10 responders, both before and after RM (Figure 3, left; before RM: 4.4 ± 1.5% in nonresponders vs 5.2 ± 2.3% in responders, not significant; after RM: 9.6 ± 4.6% in nonresponders vs 11.2 ± 4.7% in responders, not significant). The cardiac-related activities in ROI4 (ROI4_C_) were also slightly lower in the nonresponder group, both before and after RM (Figure 3, middle; before RM: 6.9 ± 1.7% in nonresponders vs 7.3 ± 1.6% in responders, not significant; after RM: 8.5 ± 3.6% in nonresponders vs 10.4 ± 4.1% in responders, not significant). The relative ventilation-perfusion index calculated with Eq. 3 in both groups showed negative values before RM, which indicates less ROI4_V_ than ROI4_C_ proportional to respective global values (Figure 3, right; −35.8 ± 20.7% in nonresponders vs -30.7 ± 17.6% in responders, not significant). The ventilation-perfusion index became positive after RM in both groups. The index was higher, but not significantly so, in nonresponders in ROI4 (9.6 ± 31.5% in nonresponders vs 3.3 ± 17.6% in responders). No significant differences were found in other ROIs after RM (ROI1: −34.2 ± 25.2% in nonresponders vs −35.9 ± 16.7% in responders; ROI2: −5.1 ± 28.4% in nonresponders vs −8.3 ± 22.2% in responders; ROI3: 14.3 ± 20.9% in nonresponders vs 15.7 ± 20.9% in responders).

We evaluated 3 EIT-derived indices that correlated with lung tissue recruitment (Table 3). In 5 out of 10 nonresponders and 9 out of 10 responders, ventilation was distributed mainly in dependent regions after RM (as indicated by a CoV index >50%). When the natural logarithms of the ΔROI4 values were considered, 8 patients among the responders and 6 patients among the nonresponders were found to exhibit a value higher than 1. Because the average distribution of ventilation in ROI4 before RM was 4.8%, a natural logarithm of ΔROI4 equal to 1 corresponded to an increase of over 50% in distribution in ROI4. Due to the mixture of patients with remarkable and insufficient lung tissue recruitment in the nonresponder group, the average value of ln(ΔROI4) was 1.3. For those patients with an insufficient mechanical response to RM, the GI index values were lowest at the highest PEEP level and increased during the decremental PEEP trial. Among the nonresponders, no statistically significant difference in ROI4_C_ was found between the patients with remarkable mechanical recruitment and those with a mechanically insufficient response (either before or after RM).

**TABLE 3:**
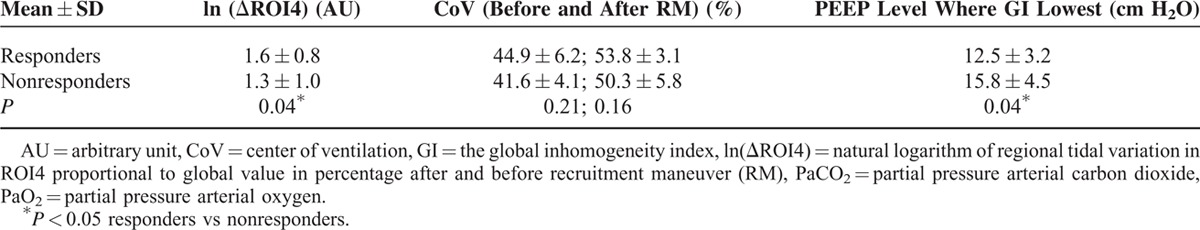
Summary of EIT-derived Indices in the Responder (PaO_2_ + PaCO_2_ ≥400 mm Hg) and nonresponder groups (PaO_2_ + PaCO_2_ <400 mm Hg)

**FIGURE 4:**
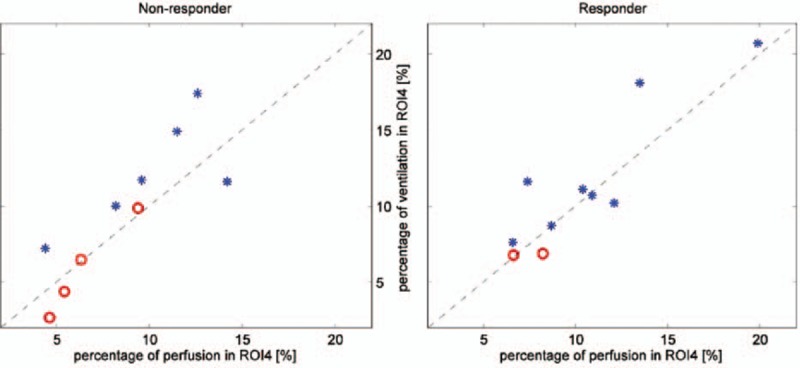
Ventilation distribution and cardiac-related activities in ROI4 after RM in the nonresponder (left) and responder (right) groups. The diagonal dashed line represents the relative ventilation-perfusion index equal to 0. Blue stars correspond to the patients who showed remarkable mechanical recruitment (ln[ΔROI4] >1). Red circles correspond to the patients with an insufficient mechanical response to RM (ln[ΔROI4]≤1). RM = recruitment maneuver, ROI = region of interest.

The ventilation distribution and cardiac-related activities in ROI4 after RM were further investigated within each group (Figure 4). Patients who displayed a relative ventilation-perfusion index close to 0 were positioned close to the diagonal line (dashed lines in Figure 4). In the nonresponder group, patients with remarkable mechanical recruitment (ln[ΔROI4] >1, blue stars in Figure 4, left) deviated from the diagonal line. A significant difference in the relative ventilation-perfusion index was found between the patients with remarkable (blue stars) and insufficient (red circles) mechanical recruitment in the nonresponder group (*P* < 0.01). In the responder group, 2 patients deviated from the diagonal line (Figure 4, right).

## DISCUSSION

In the present study, we performed RM in 20 ARDS patients under mechanical ventilation. We were able to demonstrate that not all patients exhibiting appreciably recruited lung tissue during RM showed significant improvement of oxygenation, and vice versa.

**FIGURE 5:**
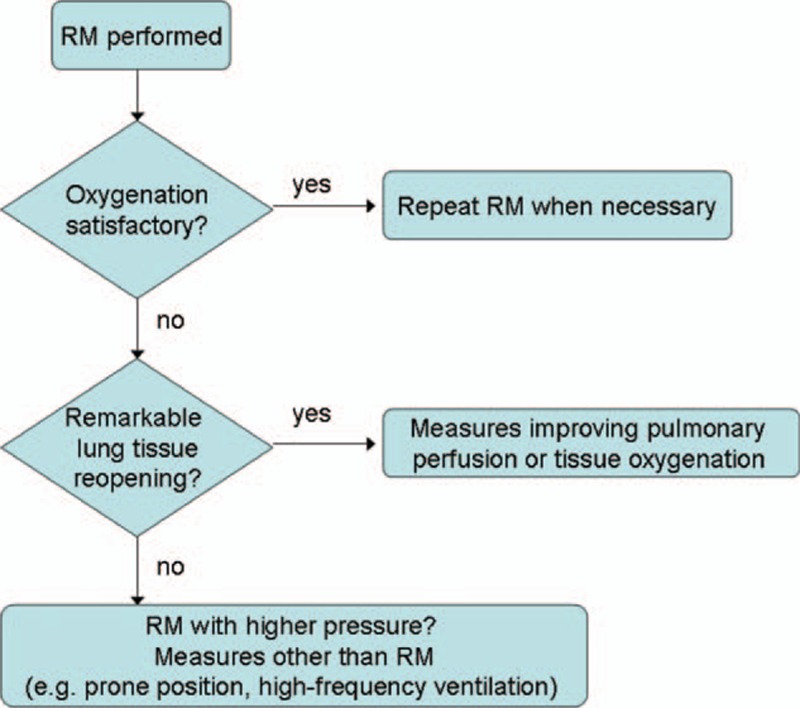
Potential workflow in clinical practice derived from the findings of this study. Please refer to the “Discussion” section for detailed implications and limitations. RM = recruitment maneuver.

According to the Berlin Definition, the severity of ARDS is defined based on the degree of hypoxemia.^27^ The aim of RM is to reopen the collapsed lung tissue and thereby improve oxygenation and reduce lung injury during tidal recruitment/derecruitment. Several recent studies have shown that EIT can be used to monitor the ventilation redistribution induced by RM,^28–30^ in accordance with the findings of the present study (Figures 1 and 2). Since the proposal of the open lung concept,^2,31^ RMs have often been used to achieve better gas exchange and improve respiratory mechanics.^3,32–34^ However, RM may introduce overdistension, and the peak airway pressure (*P*_peak_) level and the duration of RM are debatable.^21,22,35^ In the present study, we selected *P*_peak_ = 40 cm H_2_O (PEEP 20 cm H_2_O + PC 20 cm H_2_O) for the RM procedure, which was the lowest value from the recommendations of Papadakos and Lachmann.^3^ One may argue that an RM with a higher *P*_peak_ might have recruited more collapsed lung tissue and resulted in the categorization of more patients as responders. We deliberately selected this pressure level to minimize the degree of overdistension. Moreover, we considered it sufficiently high to prove our hypothesis. We found that even when lung tissue reopening was evident, oxygenation might not be satisfactory (Figure 2). In both nonresponders and responders, different types of ventilation-perfusion mismatch were found in ROI4 before and after RM (Figure 3, right). Due to lung tissue reopening, the proportion of ventilation distributed in ROI4 compared with the global value was lower than that of perfusion before RM, but higher than that of perfusion after RM. By further analyzing the ventilation and cardiac-related activities within responders and nonresponders, we observed that patients showing remarkable lung tissue recruitment in the nonresponder group exhibited a greater ventilation-perfusion mismatch (Figure 4, left). Based on the implications of these findings, a potential workflow for clinical practice is presented in Figure 5. The ideal outcome is indicated by the category “responders with remarkable mechanical recruitment.” Other outcomes are discussed below:

1. Six patients among the nonresponders displayed a natural logarithm of ΔROI4 >1, which suggested considerable reopening of lung tissues in ROI4 during RM. However, their PaO_2_ + PaCO_2_ values were still lower than 400 mm Hg, and their cardiac-related activity ratio in ROI4 deviated from ventilation (Figure 4, left). Cardiac-related activities estimated in EIT images contain information on myocardial motion and lung perfusion. The relative ventilation-perfusion index we used in the present study differed from the ratio employed in clinical practice due to the absence of absolute values in EIT measurements; nevertheless, according to this finding, we speculate that a ventilation-perfusion mismatch might be the reason for the unsatisfactory improvement oxygenation during RM. Further investigations are required to confirm this hypothesis. If this is indeed the case, raising the intrapulmonary pressure may further decrease pulmonary perfusion and increase the degree of ventilation-perfusion mismatch and overdistension. Hence, more aggressive RM procedures, which would be very unlikely to further improve oxygenation, should not be applied in these patients. Measures that improve pulmonary perfusion or tissue oxygenation should be employed in these patients (eg, increasing the cardiac output or the perfusion pressure; cardio-active agents; FiO_2_ adjustment). Because the cardiac-related signals in EIT are much weaker than the respiratory signals,^35^ the assessment of perfusion may be improved through bolus administration of saline.^36^
2. There are 2 possible explanations for those patients characterized as nonresponders with a mechanically insufficient response to RM: the pressure of RM was not sufficiently high to open the collapsed lung tissues, in which case transpulmonary pressure could be monitored; or the recruitability of the lungs was low. For the former case, RM with higher pressure could be considered. For the latter case, measures other than RM should be considered (eg, a prone position, high-frequency ventilation).
3. Two patients among the responders showed a logarithm of ΔROI4 <1, which may suggest suboptimal recruitment in ROI4. We speculated that the degree of lung injury and recruitability was low (eg, mild ARDS) in these patients. Another possible explanation is that recruitment occurred outside the EIT measurement plane, which covers a 15 to 20-cm thick cross-sectional slice of the thorax.^9^ This is certainly a limitation of EIT, which may be addressed by introducing a second measurement plane (eg, a second EIT device). However, due to possible electrical interference, measurements in 2 planes should not be performed simultaneously.

We calculated 3 different EIT-derived indices to characterize the recruitment process. Although these indices showed differences between remarkable and insufficient mechanical recruitment, we consider the logarithm of ΔROI4 to be a better index compared with the CoV and GI indices because overdistension and atelectasis were given the same weight in the calculation of the CoV and GI indices. Furthermore, ΔROI4 can be directly read from the EIT device monitor and does not require off-line analysis. The use of the natural logarithm served to magnify the small increase of ventilation in ROI4 for presentation. Simple thresholds such as ΔROI4 = 3 would deliver the same findings in the present study. The change in regional compliance in dependent regions may be calculated to indicate recruitment. Because global airway pressure is used for the calculation of regional compliance,^20^ the percent change in regional compliance in ROI4 is the same as the relative impedance change in ROI4. Another EIT-derived index—the regional ventilation delay—was proposed in a previous study to quantify intratidal recruitment/derecruitment regions during a low-flow maneuver.^37^ The ability to identify tidal recruitment during the normal ventilation mode (ie, with a much shorter inspiration time) was not clear.

One limitation of the present study was the number and variability of the patients. A previous study indicated that a longer ICU stay may result in less recruitable lung tissues,^38^ although the difference in ICU days was not statistically significant. The small number of patients included in this study (20) limited further statistical analysis. As a pilot study, the number of patients was sufficient to demonstrate that simple assessment of the ventilation-perfusion distribution at bedside using EIT may add crucial information compared with a clinical monitoring strategy employing only oxygenation parameters. To validate the findings of the present study and the workflow of the RM process (Figure 5), a prospective study of outcomes should be conducted. The threshold set during RM to distinguish responders from nonresponders (PaO_2_ + PaCO_2_ = 400 mm Hg) was also a limitation. Patients with values close to the threshold might be inappropriately classified. Similarly, the threshold of ln(ΔROI4) = 1 for distinguishing remarkable and insufficient mechanical recruitment has not been validated as more effective than other threshold values. Nevertheless, the ln(ΔROI4) index was able to reveal the heterogeneity of recruitment in the nonresponder group. Although the reliability of EIT measurements has been examined in previous studies,^13–15^ the use of CT as a reference method might further confirm these findings; however, this was not feasible in the present study due to ethical reasons. Another limitation of the present study was that a well-established reference method for lung perfusion was missing.

## CONCLUSIONS

A discrepancy in lung tissue reopening and oxygenation improvement during RM was found. EIT has the potential to evaluate the efficacy of RM by combining oxygenation measurements.
